# Removing the Age Restrictions for Rotavirus Vaccination: A Benefit-Risk Modeling Analysis

**DOI:** 10.1371/journal.pmed.1001330

**Published:** 2012-10-23

**Authors:** Manish M. Patel, Andrew D. Clark, Colin F. B. Sanderson, Jacqueline Tate, Umesh D. Parashar

**Affiliations:** 1 National Center for Immunization and Respiratory Diseases, Centers for Disease Control and Prevention, Atlanta, Georgia, United States of America; 2 London School of Hygiene and Tropical Medicine, London, United Kingdom; Menzies School of Health Research, Australia

## Abstract

**Background:**

To minimize potential risk of intussusception, the World Health Organization (WHO) recommended in 2009 that rotavirus immunization should be initiated by age 15 weeks and completed before 32 weeks. These restrictions could adversely impact vaccination coverage and thereby its health impact, particularly in developing countries where delays in vaccination often occur.

**Methods and Findings:**

We conducted a modeling study to estimate the number of rotavirus deaths prevented and the number of intussusception deaths caused by vaccination when administered on the restricted schedule versus an unrestricted schedule whereby rotavirus vaccine would be administered with DTP vaccine up to age 3 years. Countries were grouped on the basis of child mortality rates, using WHO data. Inputs were estimates of WHO rotavirus mortality by week of age from a recent study, intussusception mortality based on a literature review, predicted vaccination rates by week of age from USAID Demographic and Health Surveys, the United Nations Children's Fund (UNICEF) Multiple Indicator Cluster Surveys (MICS), and WHO-UNICEF 2010 country-specific coverage estimates, and published estimates of vaccine efficacy and vaccine-associated intussusception risk. On the basis of the error estimates and distributions for model inputs, we conducted 2,000 simulations to obtain median estimates of deaths averted and caused as well as the uncertainty ranges, defined as the 5th–95th percentile, to provide an indication of the uncertainty in the estimates.

We estimated that in low and low-middle income countries a restricted schedule would prevent 155,800 rotavirus deaths (5th–95th centiles, 83,300–217,700) while causing potentially 253 intussusception deaths (76–689). In contrast, vaccination without age restrictions would prevent 203,000 rotavirus deaths (102,000–281,500) while potentially causing 547 intussusception deaths (237–1,160). Thus, removing the age restrictions would avert an additional 47,200 rotavirus deaths (18,700–63,700) and cause an additional 294 (161–471) intussusception deaths, for an incremental benefit-risk ratio of 154 deaths averted for every death caused by vaccine. These extra deaths prevented under an unrestricted schedule reflect vaccination of an additional 21%–25% children, beyond the 63%–73% of the children who would be vaccinated under the restricted schedule. Importantly, these estimates err on the side of safety in that they assume high vaccine-associated risk of intussusception and do not account for potential herd immunity or non-fatal outcomes.

**Conclusions:**

Our analysis suggests that in low- and middle-income countries the additional lives saved by removing age restrictions for rotavirus vaccination would far outnumber the potential excess vaccine-associated intussusception deaths.

*Please see later in the article for the Editors' Summary*

## Abstract 2

A modeling analysis conducted by Manish Patel and colleagues predicts the possible number of rotavirus deaths prevented, and number of intussusception deaths caused, by use of an unrestricted rotavirus schedule in low- and middle-income countries.

## Editors' Summary

**Background:**

Rotavirus causes severe diarrhea and vomiting. It is responsible for a large number of hospitalizations among young children in developed countries (an estimated 60,000 hospitalizations per year in the US in 2005, for example). In poor countries, rotavirus is a major cause of death in children under five. In 1998, the first rotavirus vaccine, called RotaShield, was approved in the US by the Food and Drug Administration. Shortly after the vaccine became widely used, doctors noticed a small increase in a problem called intussusception among the vaccinated infants. Intussusception is a rare type of bowel obstruction that occurs when the bowel telescopes in on itself. Prompt treatment of intussusception normally leads to full recovery, but some children with the condition need surgery, and when the disease is left untreated it can be fatal. Because intussusception is a serious condition and because very few children die from rotavirus infection in the United States, the US authorities stopped recommending vaccination with RotaShield in 1999. The manufacturer withdrew the vaccine from the market shortly thereafter.

Since then, two new vaccines (named Rotarix and RotaTeq) have been developed. Before they were approved in the US and elsewhere, they were extensively tested for any adverse side effects, especially intussusception. No increase in the risk for intussusception was found in these studies, and both are now approved and recommended for vaccination of infants around the world.

**Why Was This Study Done?:**

Since 2006, hundreds of thousands of infants have been vaccinated with Rotarix or RotaTeq, with safety being closely monitored. Some countries have reported a small increase in intussusception (one to four additional cases per 100,000 vaccinated infants, compared with one per 2,000 of cases that occur in unvaccinated children). This increase is much lower than the one seen previously with RotaShield. In response to these findings, authorities in the US and other developed countries as well as the World Health Organization declared that the benefits of the vaccine outweigh the risks of the small number of additional intussusception cases in both developed and poor countries. However, because older infants have a higher risk of naturally occurring intussusception, they decided that the course of vaccination (three oral doses for Rotarix and two for RotaTeq) should be initiated before 15 weeks of age and completed before the age of 32 weeks. This is usually not a problem in countries with easy access to health facilities. However, in many poor countries where delays in infant vaccination are common, giving the vaccine only to very young children means that many others who could benefit from its protection will be excluded. In this study, the researchers examined the risks and benefits of rotavirus vaccination in poor countries where most of the rotavirus deaths occur. Specifically, they looked at the benefits and risks if the age restrictions were removed, with a particular emphasis on allowing infants to initiate rotavirus immunization even if they arrive after 15 weeks of age.

**What Did the Researchers Do and Find?:**

The researchers used the most recent estimates for how well the vaccines protect children in Africa and Asia from becoming infected with rotavirus, how many deaths from rotavirus infection can be avoided by vaccination, how many additional cases of intussusception will likely occur in vaccinated children, and what proportion of children would be excluded from rotavirus vaccination because they are too old when they come to a health facility for their infant vaccination. They then estimated the number of rotavirus deaths prevented and the number of intussusception deaths caused by vaccination in two scenarios. The first one (the restricted scenario) corresponds to previous guidelines from WHO and others, in which rotavirus vaccination needs to be initiated before 15 weeks and the full series completed before 32 weeks. The second one (called the unrestricted scenario) allows rotavirus vaccination of children alongside current routinely administered vaccines up to three years of age, recognizing that most children receive their vaccination by 1 year of life.

The researchers estimated that removing the age restriction would prevent an additional 154 rotavirus deaths for each intussusception death caused by the vaccine. Under the unrestricted scenario, roughly a third more children would get vaccinated, which would prevent an additional approximately 47,000 death from rotavirus while causing approximately 300 additional intussusception deaths.

They also calculated some best- and worst-case scenarios. The worst-case scenario assumed a much higher risk of intussusception for children receiving their first dose after 15 weeks of life than what has been seen anywhere, and also that an additional 20% of children with intussusception would die from it than what was already assumed in their routine scenario (again, a higher number than seen in reality). In addition, it assumes a lower protection from rotavirus death for the vaccine than has been observed in children vaccinated so far. In this pessimistic case, the number of rotavirus deaths prevented was 24 for each intussusception death caused by the vaccine.

**What Do These Findings Mean?:**

If one accepts that deaths caused by a vaccine are not fundamentally different from deaths caused by a failure to vaccinate, then these results show that the benefits of lifting the age restriction for rotavirus vaccine clearly outweigh the risks, at least when only examining mortality outcomes. The calculations are valid only for low-income countries in Africa and Asia where both vaccination delays and deaths from rotavirus are common. The risk-benefit ratio will be different elsewhere. There are also additional risks and benefits that are not included in the study's estimates. For example, early vaccination might be seen as less of an urgent priority when this vaccine can be had at a later date, leaving very young children more vulnerable. On the other hand, when many children in the community are vaccinated, even the unvaccinated children are less likely to get infected (what is known as “herd immunity”), something that has not been taken into account in the benefits here. The results of this study (and its limitations) were reviewed in April 2012 by WHO's Strategic Advisory Group of Experts. The group then recommended that, while early vaccination is still strongly encouraged, the age restriction on rotavirus vaccination should be removed in countries where delays in vaccination and rotavirus mortality are common so that more vulnerable children can be vaccinated and deaths from rotavirus averted.

**Additional Information:**

Please access these Web sites via the online version of this summary at http://dx.doi.org/10.1371/journal.pmed.1001330.

- The World Health Organization provides information on rotavirus
- Wikipedia has information on rotavirus vaccine and intussusception (note that Wikipedia is a free online encyclopedia that anyone can edit; available in several languages)
- The US Centers for Disease Control and Prevention rotavirus vaccination page includes a link to frequently asked questions
- PATH Rotavirus Vaccine Access and Delivery has timely, useful updates on status of rotavirus vaccines globally

## Introduction

Rotavirus infection is the leading cause of fatal diarrhea among children younger than 5 y, accounting for 453,000 deaths in the year 2008 based on recently published World Health Organization (WHO) estimates [1]. To curb this large toll of severe rotavirus disease, in 2006, the WHO recommended two rotavirus vaccines—Rotarix (GSK Biologicals) and RotaTeq (Merck & Co.)—for use in Europe and the Americas, and in 2009, they expanded this recommendation to all children worldwide [2]. These recommendations reflected the growing availability of evidence of the good efficacy profile of rotavirus vaccines—first from clinical trials in high- and middle-income countries in the Americas and Europe in 2006 and then also from low-income settings in Africa and Asia in 2009 [3]–[6].

Because a previous rotavirus vaccine (RotaShield) was found to be associated with intussusception, a rare form of bowel obstruction [7], the pivotal pre-licensure trials in the Americas and Europe for both currently available rotavirus vaccines were conducted in over 60,000 infants each to exclude this risk; these trials did not show an increase in risk of vaccine-associated intussusception similar to that found with Rotashield [3],[4]. However, recent data on the postlicensure safety of rotavirus vaccines generated from these countries has suggested a possible low level risk of intussusception (∼one to two excess cases per 100,000 vaccinated infants) in some countries but not in others [8],[9]. On the basis of considerations that this low level risk is greatly exceeded by the observed health benefits of vaccination, national and international policy and regulatory bodies have continued to support recommendations for use of rotavirus vaccine [8],[9].

In 2009, WHO recommended that rotavirus vaccines should not be initiated for infants aged 15 wk or older, with all doses being completed by 32 wk [2]. These age restrictions were driven by concerns about intussusception risk. Natural intussusception rarely occurs before 3 mo of age and the incidence increases 10-fold between 3 and 6 mo of age [10]. Therefore, a constant vaccine-associated relative risk (RR) of intussusception, particularly with the first vaccine dose that has been primarily associated with risk, would translate to more excess cases if infants were vaccinated late, beyond 3 mo of age. Similar findings were observed in the United States after use of RotaShield, prompting a debate about whether restriction of RotaShield to infants younger than 3 mo of age would have averted withdrawal of the vaccine [10]–[12]. A consequence of these strict age restrictions in countries with vaccination delays is that those arriving late for immunization would potentially not have access to the benefits of rotavirus vaccination [13],[14].

To facilitate decision making, we previously undertook a scenario analysis assessing the benefits and risks of a rotavirus vaccination strategy with and without an age restriction [15]. Since this analysis, new evidence has been published on several key parameters for the scenario analysis, including data on efficacy of rotavirus vaccines in Africa and Asia [5],[6], the effect of rotavirus vaccines on diarrhea deaths [16],[17], postlicensure data on risk of intussusception with current rotavirus vaccines [8],[9],[18], the release of updated estimates of rotavirus mortality by WHO [1] and age distribution of rotavirus disease by week of age [19], and updated data on timeliness of vaccination coverage in low- and middle-income countries [20]. The availability of these new data and the imminent introduction of rotavirus vaccines in many developing countries in Africa during the next 2 y prompted us to revise our previous analysis to provide policy makers with the most up-to-date evidence to inform decisions of best approaches to global implementation of rotavirus vaccines.

## Methods

We focused this analysis exclusively on the benefits of rotavirus mortality reduction and potential risk of fatal intussusception in children <5 y of age in 158 low- and middle-income countries with a birth cohort of 123.6 million where 99.9% of the global rotavirus mortality occurs. To explore the effect of age restriction in different parts of the world, we grouped these countries on the basis of child mortality rates, according to WHO mortality strata [21], and assigned to one of four groups: group B and C (countries with low child mortality), group D-Americas (countries in the Americas with high child mortality), group D-Asia (countries in Asia with high child mortality), and group D & E-Africa (countries in Africa with high child mortality). Because group A countries with very low child mortality (i.e., high-income) represent <0.1% of the global rotavirus deaths, they were excluded from this analysis.

### Vaccination Strategies and Coverage Estimates

For both immunization strategies, restricted and unrestricted, we assumed that rotavirus vaccine would be given at the same time as the diphtheria-tetanus-pertussis (DTP) vaccine and that vaccine coverage in the individual countries would be equal to the proportion of infants receiving each of the three DTP doses by week of age (i.e., proportion vaccinated, ρ*_v_*) during the first 3 y of life. Under the restricted schedule, if infants received their first DTP dose by ≤14 wk of age, we assumed they would receive all doses up to 32 wk of age, but if they first appeared after 14 wk, they would remain unvaccinated. On the unrestricted schedule, vaccine would be administered according to the age-specific coverage rates for each of the DTP dose up to 3 y of age.

Our DTP coverage estimates are based on vaccination data from household USAID Demographic and Health Surveys (DHSs) [22] and the United Nations Children's Fund (UNICEF) Multiple Indicator Cluster Surveys (MICS) [23] that were administered in 48 countries between 1996 and 2009. To estimate coverage for countries without DHS or MICS data, overall WHO-UNICEF 2010 country-specific coverage estimates were converted into age-specific coverage rates using regression coefficients to predict lognormal curves of timeliness. These were derived from the available DHS/MICS survey data and extrapolated to countries without a survey within a WHO region and mortality stratum. Timeliness was determined by WHO sub-region and adjusted for trends between the DHS/MICS survey year and 2010 using the WHO-UNICEF 2010 best estimates for DTP coverage data, drop-out rate between DTP1 and DTP3, the target age recommended in the country schedule, and the gross domestic product per capita [24]. This process was done separately for DTP1 and DTP3. DTP2 timeliness assumed the average of the regression coefficients used for DTP1 and DTP3.

Our analysis does not allow catch-up immunization and assumes no improvement in timeliness with the introduction of rotavirus vaccine.

### Assessment of Benefits—Base Scenario

Estimated numbers of country-specific rotavirus deaths (λ*_rv_*) were obtained from WHO, using the 95% CIs to define the triangular distributions around the point estimate (Table 1) [1]. On the basis of a WHO-sponsored review of published and unpublished studies on age distribution of diarrhea mortality and rotavirus-associated hospitalizations by week of age, we predicted 1-wk gamma age distributions for the first year of life and 4-wk age categories thereafter for countries in different WHO regions [19].

**Table 1:** Estimates of rotavirus mortality and intussusception incidence by WHO mortality group.

Mortality, Incidence, and Fatality	WHO Mortality Group Estimate (Lower Bound, Upper Bound)							
	B & C		D: Americas		D: Asia		D & E: Africa	
**Rotavirus mortality**	26,700	(24,000–29,000)	5,300	(4,600–5,900)	188,300	(160,000–217,000)	232,500	(198,000–268,000)
**Intussusception incidence (range)**	53.3	(17.7–88.2)	53.3	(17.7–88.2)	53.3	(17.7–88.2)	53.3	(17.7–88.2)
**Intussusception case fatality**	5%	(4–6)	10%	(8–12)	25%	(20–30)	25%	(20–30)

Rotavirus vaccine efficacy (ε*_rv_*) against fatal rotavirus disease was estimated from clinical trials or vaccine effectiveness studies in each WHO region (Tables 1–2) [3],[5],[6],[25]–[29]. Because efficacy against rotavirus mortality could not be directly measured in the trials, we applied efficacy estimates against the most severe rotavirus disease outcome reported in the study [3],[5],[6],[25]–[29]. This approach was reasonable given that three nationwide studies from Latin America have documented reductions in diarrhea deaths after vaccine introduction that has approximated reductions based on the efficacy of these vaccines against severe rotavirus disease [16],[17],[30]. Because both rotavirus vaccines have performed similarly in clinical trials, we assumed the same overall efficacy for the two-dose Rotarix and the three-dose RotaTeq vaccine. The efficacy parameters were age-stratified (<1 y and >1 y of age) because studies have documented lower efficacy among children older than 1 y of age [5],[25],[27]. Efficacy of partial vaccination (first dose) was also available from one country in the B & C region [27], and one country in the D-Americas region [25], but not for D-Asia and D & E-Africa. We therefore reduced the point estimates for full vaccine efficacy for Asia and Africa by the same proportion as the relative difference in efficacy between the full and partial series in D: Americas region. We used 95% CIs from the respective studies to define the beta distribution around the vaccine efficacy point estimates.

**Table 2:** Estimates of efficacy for partial and full series of rotavirus vaccine against the most severe reported outcome of rotavirus gastroenteritis, by WHO mortality group.

WHO Mortality Group	Reference	Location	Outcome	Vaccine Efficacy^a^			
				<1 y of Age		1 y of Age	
				Percent	(95% CI)	Percent	(95% CI)
**Full series efficacy during first year of life**							
B & C^b^	[26]	Latin America	≥19	97	84–100	97	84–100
D: Americas^b^	[25]	Nicaragua	≥15	77	39–92	77	39–92
D: Asia/D & E Africa	[5],[6],[29]	Bangladesh, Vietnam, Ghana, Kenya, Mali	≥15	67	37–84	34	−16 to 63
D & E Africa^c^	[28] b	South Africa & Malawi	≥11	61	44–73	—	—
**Partial series efficacy**							
B & C	[27]	El Salvador	Hospitalizations	51	26–67	51	26–67
D: Americas	[25]	Nicaragua	Hospitalizations	55	22–74	55	22–74
D: Asia/D & E Africa^d^	***—***	***—***	***—***	48	30–68	24	0–51

a Because vaccine efficacy against rotavirus deaths was not available, the model input was efficacy against the most severe reported form of rotavirus gastroenteritis in the clinical trial (≥11 denotes “severe” diarrhea and ≥15 denotes “very severe” diarrhea on 20 point Vesikari clinical scoring system).

b No decline in efficacy by age was reported by age for the very severe outcome, thus the efficacy estimate for children <2 were applied to both age groups <1 and 1 y of age.

c This trial measured efficacy during the first year of life. No estimates of efficacy were available against very severe disease that would serve as a better proxy for death (i.e., Vesikari ≥15) at these sites in Malawi and South Africa. Consistent with all other rotavirus efficacy trials where positive correlation exists between efficacy and severity score, it was assumed that efficacy in South Africa and Malawi would be higher against Vesikari score ≥15 than Vesikari ≥11. For the model, estimates of efficacy against “very severe” rotavirus diarrhea were from sites in Africa and Asia where these data were available [5],[6],[29].

d Because no partial vaccine efficacy estimates were available for Africa and Asia, we assumed that a proportional difference in efficacy between full and partial vaccination that was observed in high mortality country of Nicaragua [25].

The number of rotavirus deaths prevented was obtained from λ*_rv_*ε*_rv_*ρ*_v_*, where λ*_rv_* is the number of rotavirus deaths by week of age, ε*_rv_* is the vaccine efficacy, and ρ*_v_* is the proportion vaccinated by week of age.

### Assessment of Risk—Base Scenario

Risk of intussusception has been documented after postlicensure use of Rotarix and RotaTeq in four different studies [8],[9],[31],[32]. Each of these studies identified an approximate 4- to 6-fold increase in risk relative to background during the first week after dose 1 (Table 3), a magnitude of risk that would not have been detected in the clinical trials. No effect modification of risk with age at vaccination was reported in these studies, but the first dose of vaccine was largely administered before 15 wk. In two additional countries, no risk of intussusception was identified after the first vaccine dose [9],[18]. Risk of intussusception was not identified after the first dose in Brazil (RR = 1.1; 95% CI = 0.3–3.3) or the United States (RR = 1.2; 95% CI = 0.03–6.8). However in view of the wide CIs, particularly in the United States, a risk of small magnitude similar to that detected in the other four studies cannot be excluded [9],[18]. In Brazil, a statistically significant 2-fold risk was also identified in the first week after dose 2.

**Table 3:** Pooled estimates of risk after doses 1 and 2 of rotavirus vaccine.

Country	Reference	Rotavirus Vaccine	RR	Lower 95% Limit	Upper 95% Limit
**Dose 1**					
Australia	[8]	Pentavalent	3.9	1.5	9.9
Australia	[8]	Monovalent	4.1	1.3	13.5
Mexico	[9]	Monovalent	5.3	3	9.3
Mexico	[31]	Monovalent	6.5	4.2	10.1
Global reporting	[32]	Monovalent	5.0	1.7	14.3
**Pooled estimate** a			**5.5**	**4.1**	**7.5**
**Dose 2**					
Mexico	[9]	Monovalent	1.8	0.9	3.8
Mexico	[31]	Monovalent	1.3	0.8	2.1
Brazil	[9]	Monovalent	2.6	1.3	5.2
**Pooled estimate** a			**1.7**	**1.2**	**2.4**

a We used the weighted average of the logarithm of the RR, ∑log(RRi)ωi/∑ωi, where weight (ωi) for each study is the inverse of the variance computed from the reported 95% CIs [33]. The variance of the weighted average log RR is the inverse of the sum of each weight (1/∑ωi) and was used to compute the 95% CIs for the pooled risk estimate.

We obtained dose-specific pooled estimates of RR from each of the regions where some increase in RR of intussusception was identified (Table 3). To err on the side of risk, we excluded the US safety data from the pooled analysis because no risk was identified. For pooled estimates of vaccine-associated intussusception risk, we used the weighted average of the logarithm of the RR, ∑*log*(RR*_i_*)ω*_i_*/∑ω*_i_*, where weight (ω*_i_*) for each study [8],[9],^31^,^32^ is the inverse of the variance computed from the reported 95% CIs [33]. The variance of the weighted average log RR is the inverse of the sum of the each weight (1/∑ω*_i_*) and was used to compute the 95% CIs for the pooled risk estimate. For the uncertainty analyses, we used the 95% CIs to define the gamma distribution around the RR estimates.

The average annual incidence of natural intussusception by week of age ((λ*_is_*) was estimated from published studies. Because natural intussusception is a very rare disease, we restricted our review to studies reporting either national incidence of intussusception or incidence of intussusception from a minimum of five hospitals with known catchment population, stratified by age [34]–[51]. While intussusception incidence in this review ranged from 18–88 per 100,000 infants, the age distribution of intussusception was similar between the different studies. Thus, to obtain intussusception incidence by week of age (λ*_is_*), we applied the global intussusception incidence among infants and fit a gamma curve to intussusception surveillance data from the United States [45], the only country where intussusception incidence was available by week of age. For uncertainty analysis, parameters of the gamma curve for λ*_is_* were sampled from a normal distribution, assuming standard deviation is equal to 5% of the mid-parameter values.

Death caused by intussusception is uncommon in industrialized countries, occurring in <1% of the cases [52]. In a recently conducted national study from 16 hospitals in Mexico and 43 hospitals in Brazil (WHO group B & C), case fatality for intussusception was 1% and 5%, respectively [9]. One large study from nine countries across Africa indicated an average case fatality of about 12% [53]. No reliable estimates of case fatality were available for countries in D-Americas and D-Asia. Thus, we conservatively estimated the case fatality (δ*_is_*) to be 5% for B & C countries, 10% for D-Americas, 25% for D-Asia, and 25% for D & E-Africa. We sampled from a beta distribution, assuming standard deviation is equal to 5% of the mid parameter values to specify the upper and lower limits of δ*_is_* in uncertainty analyses.

The number of intussusception deaths associated with vaccination, during the first week after dose 1 and 2, was obtained from Bρ*_v_*[(λ*_is_*RR*_i_*) − λ*_is_*]δ*_is_*, where B is the number of births, ρ*_v_* is the proportion vaccinated by week of age, λ*_is_* is the intussusception incidence by week of age, RR*_i_* is the RR during the week after each dose, and δ*_is_* the proportion of intussusception events that lead to death.

### Sensitivity Analysis

We conducted a one-way sensitivity analysis to determine the impact on the benefit-risk ratios when assuming four conservative scenarios that would favor risk and one that would favor vaccine: (1) We assumed a relative increase of 20% in incidence and case fatality of intussusception. (2) We explored the impact of effect modification of risk by age at vaccination, by doubling estimates of RR of intussusception when dose 1 of rotavirus vaccine was administered to infants older than 14 wk of age. (3) We assumed a scenario of low vaccine efficacy by inputting the lower confidence limit for each of the efficacy estimates. (4) We explored the effect of a “pessimistic” situation combining all of the preceding three scenarios. (5) We also assessed the effect of an “optimistic” scenario of high vaccine efficacy related to factors such as possible indirect benefits or higher efficacy among children vaccinated at older ages with lesser interference of vaccine take from circulating transplacental antibodies.

### Uncertainty Analysis

The above analyses yielded estimates of rotavirus deaths averted and intussusception deaths caused under age-restricted and -unrestricted vaccination strategies. We conducted a probabilistic uncertainty analysis to assess the potential impact of simultaneous variation of each of the model inputs (λ*_rv_*, ε*_rv_*, ρ*_v_*, λ*_is_*, RR) on the precision of the benefit-risk estimates. We shifted the lognormal timeliness curves and gamma rotavirus and intussusception age curves by simultaneously sampling new shape, shift, and scale parameters for each run, with each parameter being sampled from a normal distribution with standard deviation equal to 5% of the original parameter value. On the basis of the error estimates and error distributions described for each of the model inputs, we conducted 2,000 simulations to obtain the median estimates of deaths averted and caused as well as the uncertainty ranges, defined as the 5th–95th percentile, to provide an indication of the uncertainty in the estimates. All analyses were done with Microsoft EXCEL (Microsoft Corp, 2007).

## Results

**Figure 1:**
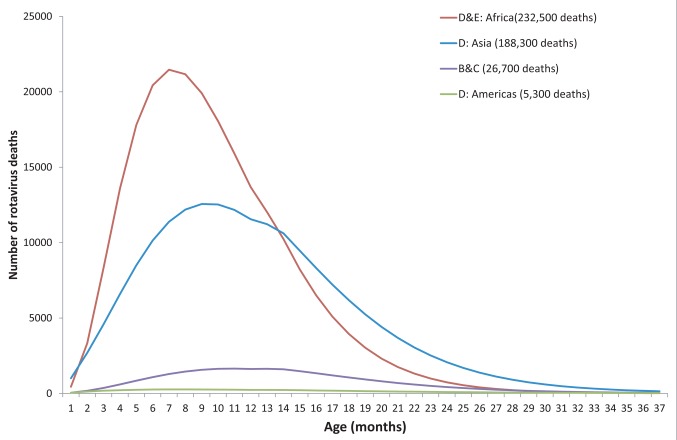
Age distribution of rotavirus deaths among children under 5 y, by WHO mortality group.

**Figure 2:**
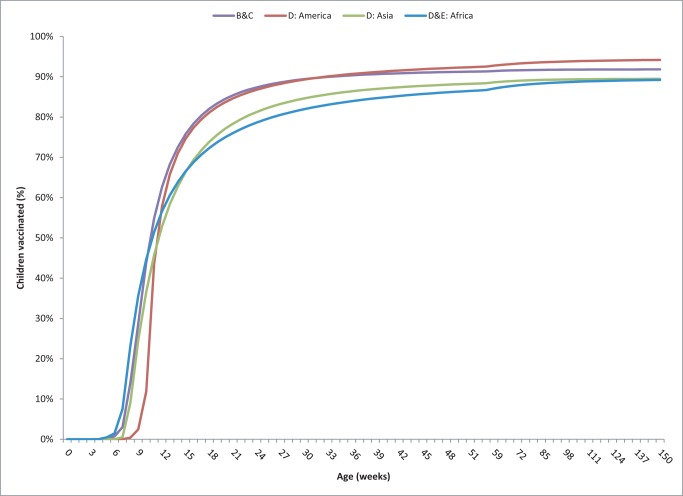
Vaccine coverage for dose 1 of DTP by week of age and WHO mortality group based on the DHSs and UNICEF MICs.

Approximately 453,000 rotavirus-associated deaths are estimated among children younger than 5 y annually without a rotavirus vaccination program (Figure 1). We project that a rotavirus vaccination program under the current age-restricted schedule would prevent almost 33% or 155,800 of these deaths (5th–95th centiles, 83,300–217,700) if delivered at the same ages at which the DTP vaccine is currently being delivered in these countries (Table 4). Without the age restrictions, a program would prevent 45% or 203,000 deaths of all rotavirus deaths (102,000–281,500), which would represent 47,200 more deaths prevented (18,700–63,700) than with an age-restricted schedule. These additional deaths prevented under an unrestricted vaccination schedule reflect an additional 18%, 21%, 25%, and 22% of the children receiving DTP1 in the WHO B & C, D-Americas, D-Asia, and D-Africa countries, respectively, compared to the age-restricted schedule in these countries (Figure 2).

**Table 4:** Rotavirus deaths averted versus excess intussusception deaths caused under age-restricted and age-unrestricted rotavirus vaccination strategies, by WHO mortality group and age.

Vaccination Strategy	Rotavirus Deaths Averted (95% CI)^a^			Intussusception Deaths Caused^a^ (95% CI)			Benefit to Risk Ratio
	Age Restriction^b^	No Age Restriction	Excess	Age Restriction^b^	No Age Restriction	Excess	
**B & C countries**							
Median	18,200	22,700	4,500	35	53	18	247
5th percentile	15,500	19,700	4,200	10	19	9	138
95th percentile	20,500	25,200	4,700	94	127	33	519
**D: Americas**							
Median	2,600	3,300	700	3	5	2	343
5th percentile	1,400	1,800	400	1	2	1	152
95th percentile	3,200	4,000	800	9	12	3	674
**D: Asia**							
Median	55,400	76,800	21,400	118	275	157	133
5th percentile	25,200	32,200	7,000	36	120	84	43
95th percentile	83,400	115,300	31,900	317	576	259	286
**D: Africa**							
Median	79,600	100,200	20,600	96	212	116	167
5th percentile	40,300	46,900	6,600	28	96	68	50
95th percentile	111,100	138,300	27,200	265	441	176	328
**All strata**							
Median	155,800	203,000	47,200	253	547	294	154
5th percentile	83,300	102,000	18,700	76	237	161	55
95th percentile	217,700	281,500	63,700	689	1,160	471	318

a Estimates of rotavirus deaths averted and intussusception deaths caused are based on efficacy, risk, and case-fatality parameters in Tables 1–3. Vaccination coverage is based on DTP vaccination rates from household DHSs and UNICEF MICSs.

b Age restriction denotes dose 1 administration by 15 wk and the full series by 32 wk of age.

**Figure 3:**
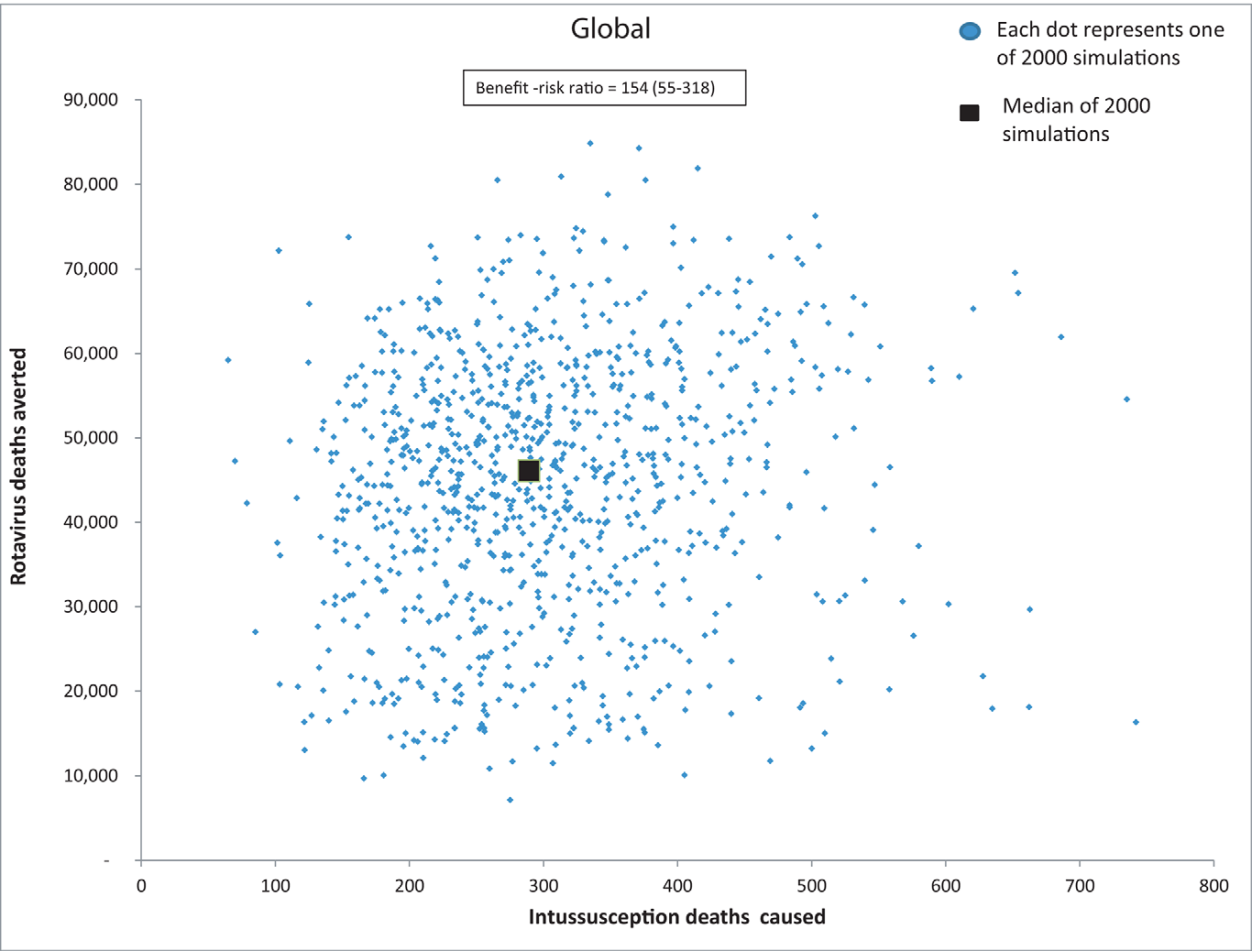
Global analysis of the relationship between esimated number of rotavirus gastroenteritis deaths avoided versus intussusception deaths caused by removal of the age restrictions for rotavirus vaccination. These estimates are from 2,000 simulations with each blue dot representing a potential estimate of rotavirus deaths prevented (y-axis) versus intussusception deaths caused (x-axis) from removal of the age restrictions given the uncertainty on the parameters in the model: rotavirus mortality, vaccine efficacy, vaccine coverage, intussusception incidence, intussusception risk from vaccine, and intussusception fatality. The black square represents the median estimate.

**Figure 4:**
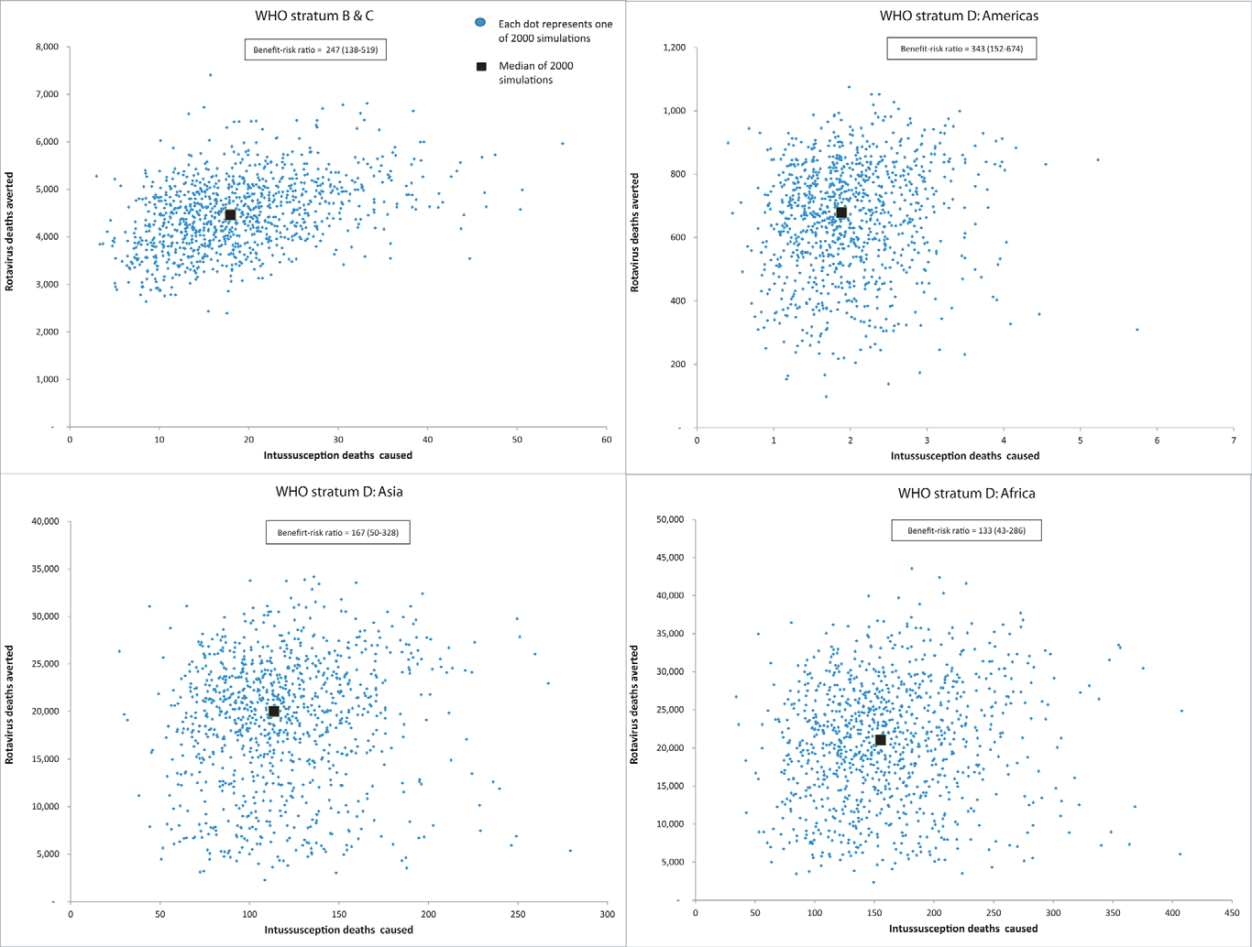
WHO region specific analysis of relationship between esimated number of rotavirus gastroenteritis deaths avoided versus intussusception deaths caused by removal of the age restrictions for rotavirus vaccination. These estimates are from 2,000 simulations with each blue dot representing a potential estimate of rotavirus deaths prevented (y-axis) versus intussusception deaths caused (x-axis) from removal of the age restrictions given the uncertainty on the parameters in the model: rotavirus mortality, vaccine efficacy, vaccine coverage, intussusception incidence, intussusception risk from vaccine, and intussusception fatality. The black square represents the median estimate. Because group A countries with very low child mortality (i.e., high-income) represent <0.1% of the global rotavirus deaths, they were excluded from this analysis.

From the perspective of risk, a rotavirus vaccination program limiting vaccination to children <15 wk of age would cause about 253 intussusception deaths (76–689) (Table 4). In contrast, a program without age restrictions would cause nearly 547 intussusception deaths (237–1,160). Thus, a vaccination policy without any age restrictions for use of rotavirus vaccines in low- and middle-income WHO countries would avert an additional 47,200 rotavirus-associated deaths and cause an additional 294 intussusception-associated deaths, compared to the current age-restricted strategy (Table 5). The median incremental benefit-risk ratio in all mortality strata was nearly 154 deaths averted for every death caused, ranging from 55–318 deaths averted for every death caused across the different mortality strata (Figures 3 and 4).

**Table 5:** Additional lives saved versus deaths caused by loosening the age restrictions for rotavirus vaccines in WHO high and very high mortality group.

Scenario	Median (5th Percentile, 95th Percentile)					
	Lives Saved		Deaths Caused		Benefit/Risk Ratio	
Base^a^	47,200	(18,700–63,700)	294	(161–471)	154	(55–318)
Base+higher intussusception rate and case fatality^b^	47,200	(18,700–63,700)	423	(232–678)	107	(38–221)
Base+increase RR with age at dose 1^c^	47,200	(18,700–63,700)	603	(174–946)	75	(27–143)
Base with low vaccine efficacy	20,400	(8,500–4,300)	294	(161–471)	71	(24–159)
Pessimistic^d^	14,400	(7,400–28,300)	703	(459–1,042)	24	(9–51)
Optimistic (Base+high vaccine efficacy)^e^	65,800	(39,900–77,000)	294	(161–471)	220	(116–407)

a Assumes point estimates for vaccine efficacy and intussusception risk and case-fatality estimates presented in Tables 1–3.

b Assumes 20% relative increase in incidence and case fatality of intussusception compared to base scenario.

c Assumes a doubling of RR of vaccine associated risk of intussusception among children receiving dose 1 beyond 15 wk of age.

d Pessimistic scenario assumes base scenario with: (1) 20% increase in background incidence and case fatality of intussusception compared to base scenario; (2) doubling of relative among children vaccinated with dose 1 beyond 15 wk of age; and (3) lower 95% confidence limit for vaccine efficacy.

e Optimistic scenario assumes the upper confidence limit for vaccine efficacy in each setting.

Under the scenarios of effect modification of risk with age at vaccination and increased incidence and case fatality of intussusception, an unrestricted schedule would cause 603 (174–946) and 423 (232–678) excess deaths, respectively, while averting about 47,200 rotavirus deaths (18,700–63,700) (Table 5). A scenario where efficacy approximated the lower confidence limit in the clinical trials would avert an additional 20,400 rotavirus deaths (8,500–34,300) under an unrestricted schedule. With pessimistic assumptions of high intussusception incidence and case fatality, high risk, and low efficacy, a vaccination program without age restrictions would cause 868 intussusception deaths (506–1,362) while preventing 20,400 rotavirus deaths (8,500–34,300), for a benefit-risk ratio of 24. In contrast, the benefit-risk ratio would approximate 220 (116–407) under an optimistic scenario of high vaccine efficacy.

## Discussion

Our analysis demonstrates that if first dose of rotavirus vaccine is restricted to children 14 wk of age or younger, rotavirus vaccines would prevent about 155,800 of the 453,000 rotavirus deaths occurring in children <5 y of age annually worldwide while resulting in 253 intussusception deaths. While most of the gap in preventable rotavirus deaths is due to the moderate efficacy of the vaccines in high mortality settings, the current age restrictions on rotavirus vaccination also contribute by potentially excluding nearly 21%–25% of the world's children, those with the highest risk of rotavirus mortality, from receiving these vaccines. Lifting the age restriction for the first dose of rotavirus vaccination would save an additional 47,200 lives yearly and would result in an additional 294 intussusception deaths, for an incremental benefit of saving 154 lives for each excess intussusception death caused.

In the past 5 y, with the introduction of rotavirus vaccines in nearly 30 countries worldwide, substantial experience has been gained with regard to the safety and effectiveness of these vaccines in the real-world setting, including against deaths [8],[9],[16]–[18],[25],[27],[54],[55]. Moreover, clinical trials for these vaccines have documented their efficacy in target populations of Asia and Africa, where majority of the rotavirus deaths occur. Given these encouraging data, the ability of the vaccines to reach children with the highest mortality will be a major determinant of their life-saving impact.

Our base estimates are conservative, erring on the side of overestimating vaccine risk for four reasons. First, over 45 publications have documented remarkable declines in severe diarrhea and rotavirus disease, including deaths, since their introduction in national immunization programs worldwide [55]. Many of these studies from different locations have demonstrated significant declines in unvaccinated members of the community, indicating indirect benefits of vaccination that we did not account for in our analysis [56]–[59]. Second, because of interference from circulating transplacental antibodies during the first several months of life, immune response to vaccine and thus efficacy is likely to be higher when children are vaccinated at older ages. For example, anti-rotavirus IgA geometric mean titers for Vietnamese infants vaccinated against rotavirus at 9 and 13 wk were lower (77 U/ml) compared to infants vaccinated at 9 and 17 wk of life (176 U/ml) [60]. Third, we assumed that some risk of intussusception exists following each of the first two doses of rotavirus vaccine in all countries worldwide; however, risk of intussusception has varied by setting, and robust studies in two large countries have not identified risk after dose 1 [9],[18]. Fourth, even in our base scenario, we assumed high rates of intussusception case fatality in all WHO regions, about 2-fold higher than those reported in the literature.

On the other hand, the benefit-risk ratios might be inflated due to several factors. First, our base scenario assumes that the risk of intussusception relative to background does not increase with age. After the withdrawal of RotaShield, a debate persisted with regard to whether the RR of intussusception might have been higher for infants vaccinated beyond 14 wk of age [11],[12]. While limited data from an evaluation in Mexico does not suggest effect modification of risk by age for current vaccines [9], we incorporated a scenario of increased risk with age at vaccination that indicated that vaccination would avert 75 rotavirus deaths for each excess intussusception death. Second, our model might have overestimated vaccine coverage among children at the highest risk of dying from rotavirus as these might be the hardest to reach, thus inflating the mortality benefits of vaccination relative to the risks in our model. However, data from Mexico and Brazil, where substantial reductions in diarrhea deaths have occurred in all regions of both countries after the introduction of vaccine [16],[17], provides some reassurance that vaccine is reaching those at the highest risk of dying.

While the numerical benefits of relaxing the age restriction on rotavirus vaccination exceed the risks, other factors are relevant for policy considerations. First, the age restrictions for rotavirus vaccines potentially offer an incentive to improve timeliness of vaccination, which would potentially have far reaching benefits beyond just prevention of rotavirus disease. However, reasons for delays in vaccination in developing countries are complex and it is not known if a policy of restricting the first dose of rotavirus vaccines alone would be a sufficient motivational factor for parents and countries to improve timeliness of vaccination. Indeed, some delays may be due to unavoidable factors, such as contraindications. Second, while the unrestricted vaccination scenario allows for vaccination at any age during the first 3 y of life, few children arrive for vaccination beyond 1 y of life. It is important to note that delays in vaccination particularly beyond 1 y of life will reduce benefits substantially because of increasing probability of acquiring natural immunity from wild-type rotavirus infection. Third, a death caused by an intervention may be perceived worse than a death caused by a failure to intervene [61]–[63]. However, some evidence suggests that individuals may regret disease resulting from withholding vaccine as much as side effects from vaccination [63]. Furthermore, after the RotaShield experience, ethicists argued equal culpability for deaths caused by withholding the vaccine as for deaths resulting from the vaccine [64]. Finally, our analysis did not address high income countries where mortality from both rotavirus disease and from intussusception is uncommon, and thus the benefit-risk considerations will differ. Furthermore, vaccination is more timely in these settings (e.g., in the United States, 93% of the DTP1 is given by 15 wk of age [65]), and thus decisions will likely have to be made at a country level based on evaluation of local data.

In summary, using emerging, real-world data on rotavirus and intussusception mortality and rotavirus vaccine efficacy, safety, and coverage, we estimate that removing the age restrictions on rotavirus vaccination would avert 47,200 additional rotavirus deaths in low- and middle-income countries. In April 2012, WHO's Strategic Advisory Group of Experts reviewed the evidence presented in this paper and recognized that the 15-wk and 32-wk age restrictions for rotavirus vaccines are preventing vaccination of many vulnerable children [66]. SAGE encourages timely vaccination, but no longer universally recommends the age restrictions, supporting their removal in seetings where mortality benefits outweigh the risk so that many thousands more deaths would be averted and immunization programs are able to immunize children who are currently excluded from the benefits of rotavirus vaccines. Age restriction policies will ultimately be decided at country level, but this analysis has shown a clear case for a change in policy that will be particularly instrumental for saving lives in settings where mortality from rotavirus is high and delays in timing of vaccination are common.

## Abbreviations

DHS: Demographic and Health Survey
DTP: diphtheria-tetanus-pertussis
MICS: Multiple Indicator Cluster Survey
RR: relative risk
UNICEF: United Nations Children's Fund
WHO: World Health Organization
